# Balanced engagement of activating and inhibitory receptors mitigates human NK cell exhaustion

**DOI:** 10.1172/jci.insight.150079

**Published:** 2022-08-08

**Authors:** Jacob A. Myers, Dawn Schirm, Laura Bendzick, Rachel Hopps, Carly Selleck, Peter Hinderlie, Martin Felices, Jeffrey S. Miller

**Affiliations:** Division of Hematology, Oncology and Transplantation, Department of Medicine, University of Minnesota, Minneapolis, Minnesota, USA.

**Keywords:** Immunology, Therapeutics, Anergy, Cancer immunotherapy, NK cells

## Abstract

NK cell exhaustion is caused by chronic exposure to activating stimuli during viral infection, tumorigenesis, and prolonged cytokine treatment. Evidence suggests that exhaustion may play a role in disease progression. However, relative to T cell exhaustion, the mechanisms underlying NK cell exhaustion and methods of reversing it are poorly understood. Here, we describe a potentially novel in vitro model of exhaustion that uses plate-bound agonists of the NK cell activating receptors NKp46 and NKG2D to induce canonical exhaustion phenotypes. In this model, prolonged activation resulted in downregulation of activating receptors, upregulation of checkpoint markers, decreased cytokine production and cytotoxicity in vitro, weakened glycolytic capacity, and decreased persistence, function, and tumor control in vivo. Furthermore, we discovered a beneficial effect of NK cell inhibitory receptor signaling during exhaustion. By simultaneously engaging the inhibitory receptor NKG2A during activation in our model, cytokine production and cytotoxicity defects were mitigated, suggesting that balancing positive and negative signals integrated by effector NK cells can be beneficial for antitumor immunity. Together, these data uncover some of the mechanisms underlying NK cell exhaustion in humans and establish our in vitro model as a valuable tool for studying the processes regulating exhaustion.

## Graphical abstract

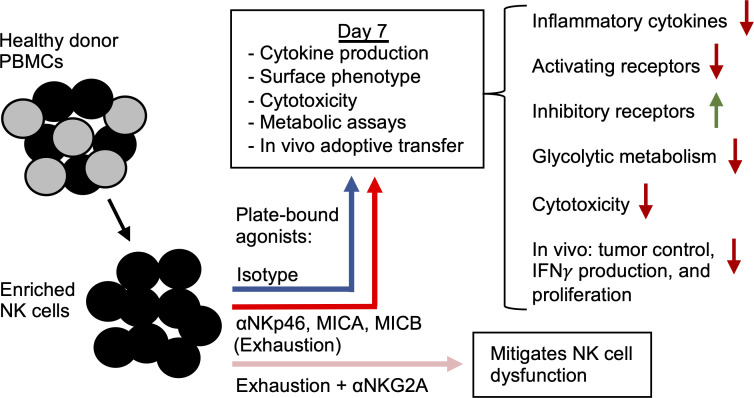

## Introduction

Due to their potent antitumor abilities, NK cells have been a major focus of cancer immunotherapy in recent years. Although randomized controlled trials of NK cell–based therapies are still needed to substantiate efficacy, multiple clinical trials have reported that these treatments are safe and effective for some cancers, especially hematologic malignancies (1, 2). Nevertheless, several factors limit the strength of these therapies. One major hindrance to clinical efficacy is immune exhaustion, a dysfunctional phenotype brought on by prolonged lymphocyte activation. Although exhaustion has been relatively well characterized in T cells, the mechanisms underlying NK cell exhaustion, and rescue therefrom, remain poorly understood, because of several complicating factors. Indeed, there appear to be multiple states of NK cell dysfunction (e.g., anergy, senescence, suppression) with phenotypes, functional profiles, and etiologies that overlap with exhaustion (3). Furthermore, NK cell exhaustion can be induced via several distinct processes, including chronic exposure to cytokines (4), repeated killing of class I MHC^–^ tumor cells (5, 6), chronic viral infection (7), and homeostatic expansion following hematopoietic stem cell transplant (8), which poses a challenge when seeking to identify unifying molecular mechanisms of exhaustion. Nevertheless, the common feature throughout these populations is loss of one or more effector functions, which ultimately defines exhaustion. Given the ambiguous boundaries that separate these states of dysfunction and the gaps of knowledge surrounding NK cell exhaustion, there is an urgent need to better define the phenotypes and functional traits that define exhaustion as well as the mechanisms through which this state is brought about.

Existing data in this area suggest that NK cell exhaustion is primarily mediated by prolonged exposure to cytokines and excessive stimulation by transformed or infected cells. When mice are repeatedly injected with IL-2, murine NK cells exhibit decreased cytotoxicity and IFN-γ production, downregulation of Eomes, downregulation of activating receptors, and upregulation of inhibitory receptors (7). Human NK cells that are chronically exposed to IL-15 in vitro display similar defects in tumor target killing and cytokine production as well as decreased phospho-signaling and significantly diminished oxidative metabolic function (4). Several groups have reported exhausted NK cells in murine tumor models that display similar phenotypes. Common features of NK cell dysfunction in these studies include decreased cytokine production and degranulation, weakened tumor killing, and upregulation of inhibitory receptors (5, 6, 8). Interestingly, multiple groups have reported that this dysfunction is dependent upon an absence of MHC expression on tumor cells. Indeed, when NK cells are exposed to MHC-sufficient tumors, dysfunction is avoided, although these populations also fail to control tumor growth (5, 6). These latter 2 studies highlight a potential role for inhibitory signaling in averting hyporesponsiveness. In light of these data, direct study of NK cell signal balancing in the context of tumor immunology is of crucial importance. However, to our knowledge, there is scant information on this topic. Therefore, we sought to better understand how the balance of positive and negative signaling during NK cell tumor killing affects both immediate function and long-term responsiveness.

We hypothesize that persistent activating receptor stimulation induces lasting functional effects and that by tempering positive NK cell signaling with modest inhibitory stimuli, it will be possible to prevent exhaustion while maintaining sufficient signaling for proper function during activation. Our data indicate that prolonged activation of receptors NKG2D and NKp46 simultaneously resulted in NK cell exhaustion characterized by upregulation of checkpoint markers, downregulation of activating receptors; decreases in cytokine production, degranulation, metabolic function, and target cell killing; and decreased in vivo persistence, function, and tumor control. Moreover, we demonstrate that tempering positive signals from NKG2D and NKp46 with simultaneous stimulation through NKG2A mitigated several aspects of exhaustion, rendering NK cells more responsive to target cell stimulation. These results indicate that balancing signals of antitumor NK cells is critical for maintaining responsiveness to targets and has implications for checkpoint blockade approaches and their auxiliary enhancement therapies.

## Results

### Prolonged stimulation through NKG2D and NKp46 functionally exhausts human NK cells.

**Figure 1:**
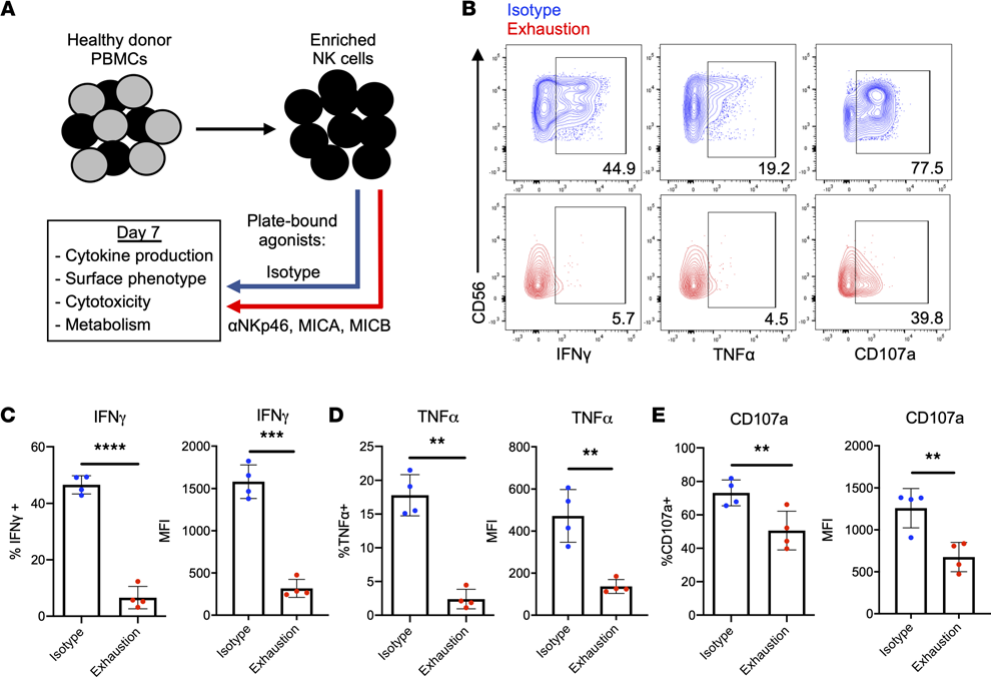
Prolonged stimulation through activating receptors induces NK cell exhaustion. (**A**) Schematic representing the in vitro model of exhaustion. Agonists of NKp46 (anti-NKp46) and NKG2D (MICA and MICB) were adsorbed onto tissue culture plates and used to stimulate NK cells for 7 days. Plate-bound isotype IgG served as a control. Both groups received 1 ng/mL IL-15. (**B**) NK cells harvested from isotype-coated and exhaustion plates (day 7) were incubated with K-562 targets for 4 hours (E/T: 2:1). Cytokine production (IFN-γ and TNF-α) and degranulation (CD107a) were measured via flow cytometry. Isotype NK cells (top row) in blue, exhausted NK cells (bottom row) in red. E/T, effector/target. (**C**–**E**) Quantification of cytokine production and degranulation as percentage of parent population (live, CD3^–^CD56^+^ cells) and mean fluorescence intensity (MFI) (*n* = 4). Paired *t* tests were used for comparisons. **P < 0.01; ***P < 0.001; ****P < 0.0001.

To model exhaustion, plate-bound agonists of the activating receptors NKp46 and NKG2D were used to stimulate NK cells (Figure 1A). It is likely that NK cells encounter ligands for multiple activating receptors during tumor surveillance. Therefore, because these receptors are known to mediate tumor killing (9–11), these conditions are suitable for modeling exhaustion in vitro. Moreover, NKG2D has been implicated in mediating activating receptor–induced dysfunction in murine and human NK cells (7, 12). In agreement with these studies, when restimulated with K-562 target cells following stimulation, exhausted NK cells produced markedly less IFN-γ and TNF-α than NK cells receiving only IL-15 over the same 7-day period (Figure 1, B–D). Furthermore, these cells degranulated significantly less than their unstimulated counterpart, suggesting potential defects in target killing (Figure 1, B and E). Similar results were observed when NK cells were stimulated for 3 days. However, no significant differences in degranulation were observed at this time point (Supplemental Figure 1, A–C; supplemental material available online with this article; https://doi.org/10.1172/jci.insight.150079DS1). When stimulated through NKG2D and NKp46 individually, NK cells also became functionally exhausted, though not to the same degree as cells stimulated through both receptors (Supplemental Figure 1, D–F). Exhausted and nonexhausted NK cells exhibited similar degrees of viability and apoptosis, supporting the notion that cell death was not significantly affecting the function of exhausted NK cells (Supplemental Figure 2, B–E). Interestingly, prolonged stimulation through activating receptors also dramatically downregulated expression of CD56, resulting in an accumulation of CD56^–^ NK cells — a phenotype observed in multiple clinical cases of NK cell dysfunction (Supplemental Figure 2, F–I) (13–15).

**Figure 2:**
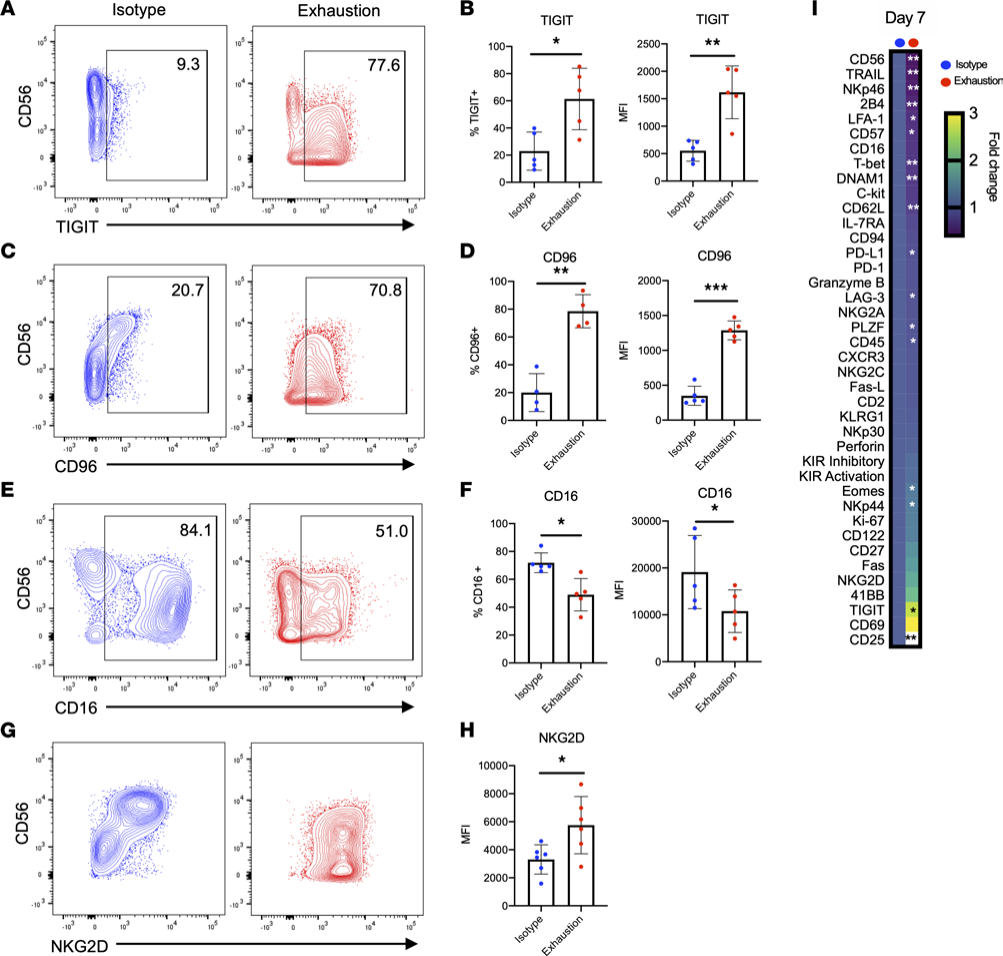
Prolonged stimulation through activating receptors alters expression of activating and inhibitory receptors. (**A**–**H**) NK cells were harvested from isotype-coated and exhaustion plates (day 7), and expression of TIGIT (**A** and **B**) (*n* = 5), CD96 (**C** and **D**) (*n* = 3, *n* = 5), CD16 (**E** and **F**) (*n* = 5), and NKG2D (**G** and **H**) (*n* = 6) was measured via flow cytometry. (**I**) At day 7 of stimulation, cells from 3 donors were analyzed via mass cytometry (CyTOF). Heatmap indicates fold change relative to isotype mean metal intensity. Markers are ordered from most downregulated to most upregulated relative to control. Paired *t* tests were used for comparisons (*n* = 3). *P < 0.05; **P < 0.01; ***P < 0.001.

Assessing the surface phenotype of exhausted NK cells, we observed significant downregulation of activating receptors and upregulation of inhibitory receptors (Figure 2, B, D, F, and H), 2 hallmarks of NK cell exhaustion described extensively in the literature (5–7, 16–19). The kinetics of these changes differed for activating and inhibitory receptors. In this system, expression of the inhibitory receptors TIGIT and CD96 dramatically increased over 7 days of exhaustion (Figure 2, A–D, and Supplemental Figure 1, G–J). In contrast, NKG2D and CD16 were downregulated early (Supplemental Figure 1, K–M), and while CD16 expression remained low at day 7, NKG2D appeared to slowly increase in expression over time, matching the control group by day 7 (Figure 2, E–H). Of note, exhausted NK cells exhibited no changes in expression of programmed cell death 1 (PD-1) but upregulated T cell immunoglobulin mucin receptor 3 (Tim-3) and NKG2A (Supplemental Figure 3, A–C). To explore the phenotype of exhausted NK cells more deeply, we used mass cytometry by time-of-flight (CyTOF). Here, we confirmed the previously identified expression patterns for several markers, including CD56, TIGIT, CD96, CD16, and NKG2D, though the latter 2 did not reach statistical significance. We observed that exhausted NK cells significantly downregulated molecules involved in activation, killing, migration, and adhesion (Figure 2I), including TRAIL, NKp46, 2B4, LFA-1, and CD62-L. Interestingly, T-bet, a transcription factor known to regulate NK cell cytotoxicity, was also significantly downregulated in exhausted cells (Figure 2I). These phenotypic changes are consistent with an exhaustion signature and provide possible explanations for the observed functional defects against tumor cells.

**Figure 3:**
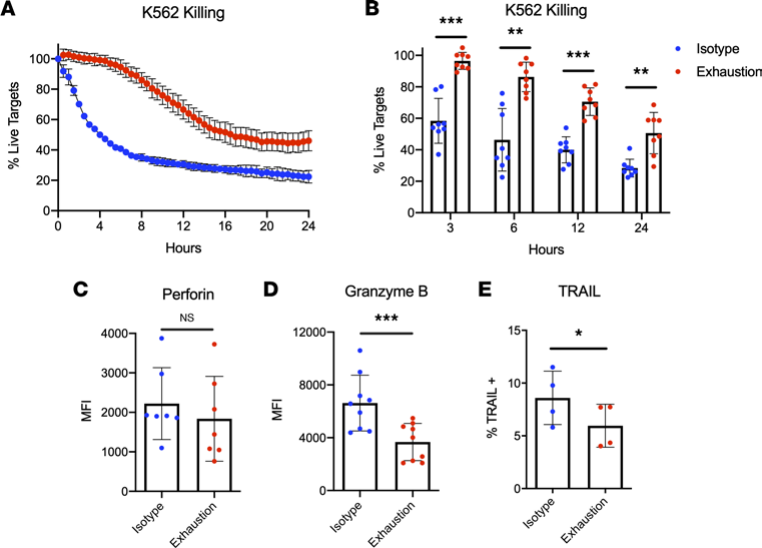
Prolonged stimulation through activating receptors weakens natural cytotoxicity. (**A**) NK cells harvested from isotype-coated and exhaustion plates (day 7) were incubated with K-562 targets for 48 hours (E/T: 2:1). K-562 cells were labeled with CellTrace Far Red, and a fluorescent caspase-3/7 reagent was added to detect apoptotic cells. Killing was measured in real time using the IncuCyte live cell killing platform. (**B**) Percentage live targets was quantified for both groups of cells at multiple time points (*n* = 8). (**C**–**E**) NK cells were harvested from isotype-coated and exhaustion plates (day 7), and expression (MFI or % of parent) for granzyme B (**C**) (*n* = 9), perforin (**D**) (*n* = 7), and TRAIL (**E**) (*n* = 4) was measured via flow cytometry at the noted time points. Paired *t* tests were used for comparisons. *P ≤ 0.05; **P < 0.01; ***P < 0.001.

To investigate whether exhausted NK cells exhibited defects in natural cytotoxicity, the IncuCyte live cell imaging system was used to visualize dynamic killing over time during NK cell coculture with targets. By tracking cytotoxicity for 24 hours during coincubation with K-562 cells, substantial killing defects were observed in exhausted NK cells harvested at day 7 (Figure 3, A and B). Because NK cell serial killing is primarily mediated by perforin and granzyme B, we hypothesized that expression of these effector molecules was significantly hampered in exhausted NK cells. After 7 days of stimulation, exhausted NK cells expressed significantly less granzyme B relative to unstimulated cells, though perforin downregulation was only apparent after 3 days of stimulation (Figure 3, C and D, and Supplemental Figure 3E). NK cells can also trigger tumor apoptosis through death receptor ligands; thus, expression of TRAIL and FasL was evaluated at day 7. Expression of these death receptors was not significantly different after 3 days of stimulation (Supplemental Figure 3, G and H), but both were significantly downregulated on exhausted cells after 7 days (Figure 3E and Supplemental Figure 3F). However, at the 7-day time point, FasL^+^ cells in both groups constituted less than 1% of the parent population (Supplemental Figure 3F). Together, these data demonstrate that prolonged stimulation through NKp46 and NKG2D results in profound NK cell exhaustion that hinders crucial aspects of innate immune function, such as proinflammatory cytokine production and natural cytotoxicity.

### Exhausted NK cells exhibit defects in glycolytic health.

**Figure 4:**
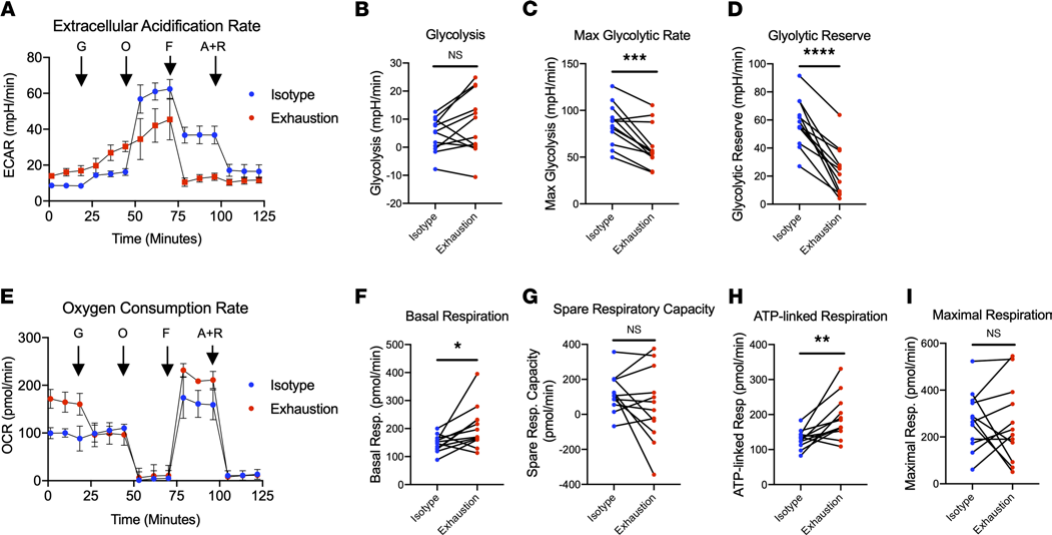
Prolonged stimulation through activating receptors alters the glycolytic and oxidative metabolism of NK cells. (**A**) NK cells harvested from isotype-coated and exhaustion plates (day 7) were immobilized on tissue culture plates and subjected to live cell metabolic assays using Agilent’s Seahorse XFe24 Analyzer. Extracellular acidification rates (ECARs) were measured following injection of glucose (G), oligomycin (O), FCCP (F), and antimycin A + rotenone (A+R). (**B**–**D**) Graphical representations of glycolysis (**B**), maximum glycolytic rate (**C**), and glycolytic reserve (**D**) are pictured (*n* = 12). (**E**) OCRs were measured following injection of glucose (G), oligomycin (O), FCCP (F), and antimycin A + rotenone (A+R). (**F**–**I**) Graphical representations of basal respiration (**F**), spare respiratory capacity (**G**), ATP-linked respiration (**H**), and maximal respiration (**I**) are pictured (*n* = 12). Paired *t* tests were used for comparisons. *P ≤ 0.05; **P < 0.01; ***P < 0.001; ****P < 0.0001.

In recent years, the importance of NK cell metabolism on antitumor function has become increasingly appreciated. Therefore, the metabolic health of exhausted NK cells was also evaluated. This question was evaluated with the Agilent Seahorse metabolic analysis platform, which sensitively detects changes in rates of oxygen consumption and extracellular acidification in live cell cultures as proxies for oxidative phosphorylation and glycolysis, respectively. Although there was no change in the average oxygen consumption rate (OCR) or spare respiratory capacity (SRC) between exhausted NK cells and the control, exhausted NK cells exhibited a significantly reduced glycolytic capacity (i.e., maximum extracellular acidification rate) and glycolytic reserve (Figure 4, A–I). Given the strict glucose requirements of NK cells (20), it is possible that these observed metabolic defects have a profound impact on the effector function of exhausted NK cells.

### Exhausted NK cells exhibit defects in persistence and function in vivo.

**Figure 5:**
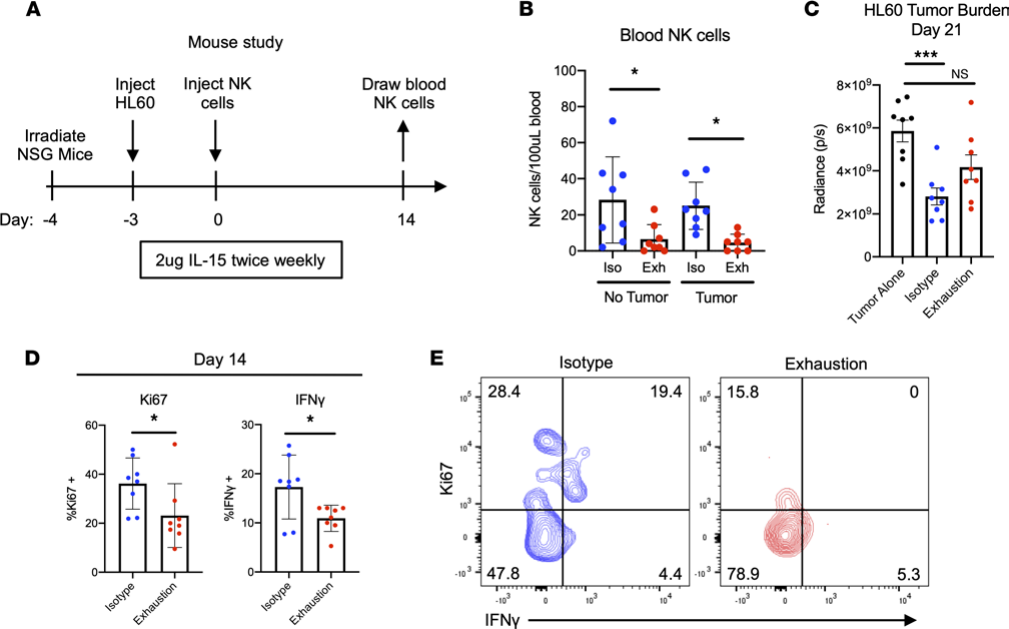
Prolonged stimulation through activating receptors results in functional and proliferative defects in vivo. (**A**) Schematic representing experimental design: sublethally irradiated NSG mice were injected (i.v.) with HL-60-Luc2 cells and injected (i.v.) with NK cells 3 days later. NK cells had been incubated for 7 days on plates with either isotype IgG (control) or anti-NKp46 and MICA/B as previously described. (**B**) Fourteen days after NK cell injection, blood was drawn, and NK cells were counted via flow cytometry. NK cells were human CD45^+^CD56^+^CD3^–^. One-way ANOVA was used for comparisons (*n* = 8). *P ≤ 0.05. (**C**) HL-60 tumor burden was tracked via bioluminescent imaging (BLI), with day 21 data pictured. One-way ANOVA was used for comparisons (*n* = 8). ***P < 0.001. (**D** and **E**) Fourteen days after NK cell injection, NK cells were restimulated with K-562 leukemia cells for 4 hours as previously described. Ki67, IFN-γ, TNF-α, and CD107a expression was assessed via flow cytometry (**D**). Paired *t* tests were used for comparisons (*n* = 8). *P ≤ 0.05. Representative data depict Ki67 and IFN-γ expression following K-562 restimulation (**E**).

Long-lived deficiencies in in vivo persistence and replication are hallmarks of lymphocyte exhaustion. Although in vitro data seemed to indicate no differences in viability and an advantage in cell expansion in the exhausted group, likely due to initial synergy between cytokine signaling and activating receptor signaling, this evaluation was limited in nature and required further physiologic determination (Supplemental Figure 2, A–C). To test whether exhausted NK cells from our model of plate-bound stimulation exhibited a true exhausted phenotype in vivo, we used a xenogeneic tumor mouse model. This system provides NK cells continued antigen stimulation (tumor) and allows us to study differences in blood NK cell numbers, NK cell function, and tumor burden in vivo. Here, sublethally irradiated NSG mice were injected (i.v.) with HL-60-Luc2 cells, injected (i.v.) with either control or exhausted NK cells 3 days later, and treated with IL-15 twice weekly (Figure 5A). In parallel, NK cells were also injected into irradiated mice *without* tumor to determine how relief from antigen stimulation affects exhaustion longitudinally (Supplemental Figure 7A). In both models, after 2 weeks, blood NK cells were drawn and restimulated in function assays with K-562 leukemia cells as previously described.

Following the blood draw, we observed that in mice with and without tumors, significantly fewer exhausted cells were present in the blood relative to control NK cells (Figure 5B). Additionally, beginning at day 21, we observed that mice receiving control NK cells had significantly lower tumor burdens than mice not receiving NK cells. However, mice receiving exhausted NK cells exhibited no significant differences in this same comparison across multiple time points (Figure 5C and Supplemental Figure 7C). When restimulated with tumor cells ex vivo, control NK cells produced significantly more IFN-γ than exhausted cells. Furthermore, relative to exhausted NK cells, a significantly larger fraction of the isotype-stimulated group was Ki67^+^ (Figure 5, D and E). Interestingly, although exhausted NK cells drawn from mice without tumors also displayed similar defects in IFN-γ production, this population did not show the same deficit in Ki67^+^ cells as was observed in tumor-bearing mice, raising the possibility that extended antigen stimulation is required for a loss of proliferative capacity (Supplemental Figure 7B). Taken together, these data demonstrate that extending the duration of chronic activating receptor stimulation in vivo results in significant defects in persistence, proliferative capacity, and cytokine production.

### Balancing signals via NKG2A engagement mitigates NK cell dysfunction.

**Figure 6:**
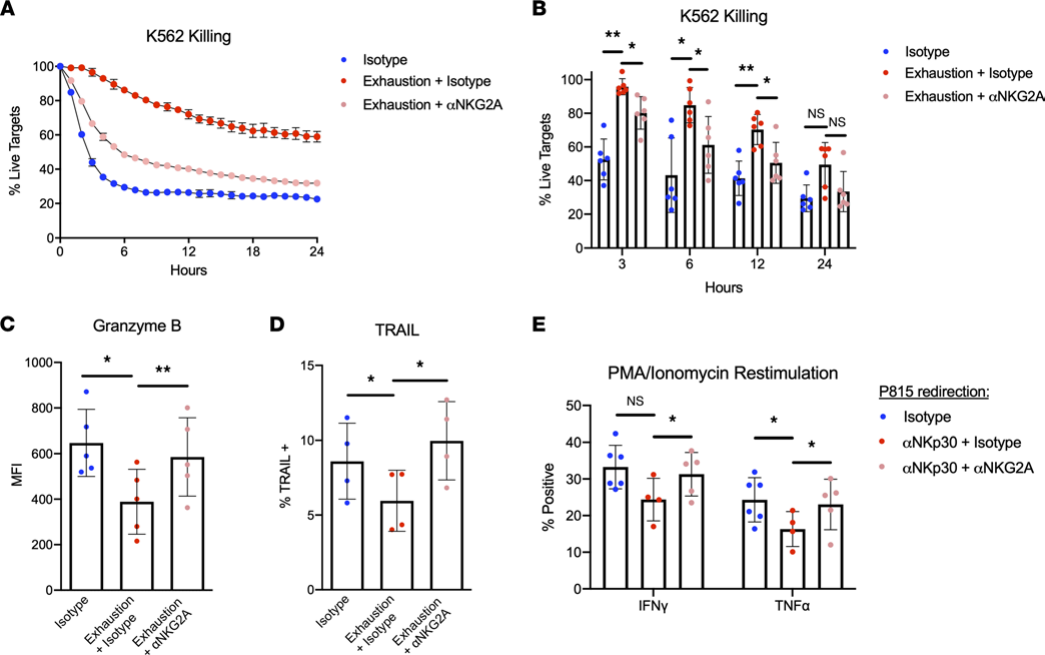
Engagement of NKG2A during exhaustion rescues defects in cytotoxicity and cytokine production. (**A**) NK cells were incubated with both positive (anti-NKp46 plus MICA/B) and inhibitory (anti-NKG2A) stimuli or with positive stimuli plus isotype IgG. NK cells plated on isotype-bound cells served as a control for stimulation through the low-affinity Fc receptor CD16. NK cells were incubated with K-562 target cells (E/T 2:1), and live cell killing assays were performed using the IncuCyte platform as previously described. (**B**) Percentage of live targets was quantified for each group of cells at multiple time points (*n* = 6). One-way ANOVA with Dunnett’s multiple comparisons was used for statistical analysis (with each time point compared independently). (**C** and **D**) Expression of granzyme B (**C**) (*n* = 5) and TRAIL (**D**) (*n* = 4) was assessed via flow cytometry. One-way ANOVA with multiple comparisons was used for statistical analysis. (**E**) Irradiated P815 cells were coated with either anti-NKp30 and anti-NKG2A or anti-NKp30 and isotype IgG. P815 coated in isotype alone served as a negative control. NK cells were incubated with P815 target cells (E/T 2:1) for 5 days and subsequently stimulated with PMA/ionomycin for 4 hours. Following stimulation, cytokine production was assessed via flow cytometry (*n* = 5). One-way ANOVA with multiple comparisons was used for statistical analysis (with IFN-γ and TNF-α compared independently). *P ≤ 0.05; **P < 0.01.

Our data thus far reveal that excessive signaling through activating receptors leads to NK cell exhaustion. Therefore, we hypothesized that balancing these stimuli with modest degrees of inhibitory signaling could prevent dysfunction. To address this idea, inhibitory signaling via NKG2A was integrated into our plate-bound agonist model of exhaustion. By adding either anti-NKG2A or isotype IgG in equal amounts to the existing solution of agonists (anti-NKp46, MICA, and MICB), which were adsorbed onto tissue culture plates, the conditions to test this hypothesis directly were created. Data generated through this system demonstrate that concurrent inhibitory signaling during stimulation significantly mitigated the defects in cytotoxicity conferred by exhaustion (Figure 6, A and B). Furthermore, these cells expressed significantly more granzyme B and TRAIL compared with exhausted NK cells, with levels roughly equivalent to those of unstimulated cells (Figure 6, C and D). This signal-balancing effect was apparent in the surface phenotype of these cells, as several markers that were downregulated in exhausted cells (i.e., TRAIL, CD56, NKp46, and T-bet) were not significantly different from the isotype group (Supplemental Figure 4, A–G). The NKG2A-tempering effect was partially observable when evaluating the proliferation of these cells, although the differences were not statistically significant (Supplemental Figure 5, A–C). Such an effect was not observed when assessing the metabolic capacities of these cells, as the “NKG2A-balanced” group exhibited similar levels of glycolysis and maximal respiration relative to the exhausted group (Supplemental Figure 5, D–G).

Next, we sought to ascertain whether this model of stimulation could be recapitulated using a cell-based system of exhaustion. To do this, we used a P815 mastocytoma model of redirected killing in which P815 cells were coated with anti-NKp30 and either anti-NKG2A or isotype IgG. As expected, NK cells exhibited robust control of tumor growth when NKp30 was the only receptor being activated (Supplemental Figure 6, A and B). When NKG2A agonists were added to this mode of stimulation, P815 killing was reduced, but not completely abrogated, indicating that this model could be used to test our hypothesis regarding signal balancing and exhaustion (Supplemental Figure 6, A and B). To test this hypothesis, irradiated P815 cells were coated with antibodies as described in the previous experiment and coincubated with NK cells for 5 days. Following this coincubation, cells were stimulated with PMA and ionomycin for 4 hours, after which cytokine production was analyzed via flow cytometry. We found that NK cells receiving signals from NKp30 alone produced lower levels of IFN-γ and TNF-α compared with those incubated with isotype-coated P815 cells (Figure 6E). Importantly, we also observed that cells receiving signals from NKG2A as well as NKp30 produced significantly more IFN-γ than their counterpart receiving only positive stimuli (Figure 6E). Taken together, these data indicate that the detrimental consequences of exhaustion can be alleviated if NK cells receive some inhibitory signals that temper excessive positive signaling.

## Discussion

During an immune response to cancer, antitumor NK cells are subjected to strong and repeated stimuli through their activating receptors, which results in exhaustion, characterized by decreased cytotoxicity and cytokine production. In recent years, multiple groups have reported the appearance of such cells in patients with chronic infections and cancer, correlating the exhaustion phenotype with negative clinical outcomes (21–25). Given this issue, elucidating the molecular mechanism that induces exhaustion and studying interventions that reverse or prevent this state are of the utmost clinical importance.

Our findings indicate that repeated stimulation through the activating receptors NKp46 and NKG2D induced NK cell exhaustion characterized by the same features observed in patients with chronic cancers and infection: decreased cytokine production, upregulation of inhibitory receptors, downregulation of activating receptors, and decreased cytotoxicity. Moreover, we found that chronic stimulation also resulted in defects in glycolytic metabolism — namely, reduced maximum glycolytic rate and glycolytic reserve. In recent years, the influence of metabolism on NK cell effector function has become increasingly appreciated and has highlighted the reliance of antitumor NK cells on glucose in particular (20, 26–28). Prolonged stimulation with IL-15 has been shown to induce robust defects in the oxidative metabolism of human NK cells but modest alterations in glycolytic metabolism (4). Our study finds the opposite: NK cells exhausted via activating receptor stimulation exhibited significant decreases in glycolytic capacity with no apparent defects in oxidative metabolism (Figure 4). Furthermore, we found that exhausted cells possessed higher basal and ATP-linked respiratory rates. To our knowledge, an increase in basal and ATP-linked metabolic rate has not been described in exhausted NK cells or exhausted T cells. In fact, exhausted T cells appear to have impaired oxidative metabolic function that blunts proliferation and effector function (29). Furthermore, our group has shown that the oxidative capacity of NK cells exhausted with IL-15 is significantly impaired (4). Given the contrast between these studies, as well as existing data demonstrating that different modes of stimulation rely on divergent metabolic pathways, it cannot be assumed that different types of exhausted NK cells are governed by the same underlying biological processes, and future work in this area should account for these nuanced differences (30). Nevertheless, targeting NK cell metabolic pathways has been shown to prevent cytokine-mediated exhaustion; therefore, it is possible that exhaustion mediated by activating receptors can be similarly modulated and is worthy of future study (4).

Given the disparities observed in different models of exhaustion, we must also consider the possibility that exhaustion exists as a continuum and that at the time points we are studying, we are observing only a snapshot of one point in this spectrum. For example, at day 7 in our model, exhausted NK cells exhibited a higher proliferative capacity than control cells (as evidenced by higher cell numbers and larger proportion of Ki67^+^ cells). However, this trend was reversed following adoptive transfer into tumor-bearing mice. Similarly, after 3 days of stimulation, exhausted NK cells degranulated at similar rates relative to control cells, but by day 7, this population degranulated significantly less. We attempted to extend our in vitro assay past 7 days to study other phenotypes within this continuum of exhaustion; however, by day 10, NK cells began experiencing IL-15–mediated exhaustion, which confounded evaluation of natural cytotoxicity receptor–mediated exhaustion at later time points (Supplemental Figure 8).

Next, because the efficacy of NK cell–based therapies for cancer relies on the persistence and function of NK cells in vivo, we sought to determine whether NK cell exhaustion persisted in mice with and without tumors. When NK cells were transferred into tumor-bearing mice, we found significantly fewer exhausted NK cells than control cells in the blood after 2 weeks. This decrease in cell numbers was accompanied by increased tumor burden, lower proportions of Ki67^+^ cells, and weaker IFN-γ production. Furthermore, we observed that mice receiving isotype-stimulated NK cells controlled tumor significantly more than exhausted cells (Figure 5C). Results from our experiment transferring NK cells into mice *without* tumors were similar, with lower blood NK cell numbers and decreased IFN-γ production. However, control and exhausted NK cells in this system had no significant differences in proportions of Ki67^+^ cells. Taken together, these in vivo experiments reveal that a 7-day period of in vitro stimulation is sufficient to drive long-term persistence and IFN-γ production defects, though a persistent antigen stimulus (i.e., tumor) may be necessary to drive a more significant loss of NK cells’ proliferation. Despite the IFN-γ defect, it remains difficult to speculate on the cell-intrinsic cytotoxic abilities of exhausted NK cells in this system. Indeed, differences in tumor burden may be more attributable to cell number than cytotoxicity, and a higher proportion of exhausted NK cells appeared to degranulate in an ex vivo function assay. However, similar degranulation rates do not necessarily mean exhausted NK cells kill as well as the control population, as we observed previously, likely due to decreased granzyme B content in these granules (Figure 3C).

The observation of early proliferation in vitro (Supplemental Figure 2A) followed by a drop in proliferation and persistence in vivo (Figure 5B) has been noted in models of chronic cytokine treatment and chronic viral infection, which suggests that exhaustion confers a fundamental dysfunctional state imprinted on NK cells following prolonged activation (7). That this pattern has emerged in 3 different models of exhaustion points toward potential changes to the epigenetic landscape of this population. This idea is further supported by data indicating that prolonged stimulation through the activating receptor NKG2C results in robust proliferation followed by genome-wide alterations in DNA methylation in adaptive NK cells (16).

Last, we sought to ascertain how exhaustion operates as a function of the net balance of positive and negative signals integrated by NK cell activating and inhibitory receptors, respectively. Multiple studies have demonstrated that class I MHC^–^ tumors are responsible for driving exhaustion and that the presence of MHC ligands prevents dysfunction by providing inhibitory signals to antitumor NK cells (5, 6). Such findings raise the possibility that there may be an optimal balance of activating and inhibitory signals that provide NK cells enough stimulus to kill tumor targets but not so much as to induce exhaustion. In this study, we used 2 models of exhaustion to test this hypothesis. First, we used the plate-bound model of exhaustion to demonstrate that simultaneous engagement of NKG2A during positive stimulation partially protected NK cells from the detrimental effects of prolonged activation, as evidenced by increases in cytotoxicity against leukemia targets and heightened expression of the effector molecules granzyme B and TRAIL, as well as proliferative index. Our CyTOF data also indicate other “signal-tempering” effects on phenotype, as evidenced by increases in NKp46, DNAM-1, and T-bet expression. Second, we used a P815 mastocytoma-redirected lysis model of exhaustion to show that engagement of NKG2A during a prolonged incubation with target cells can protect NK cells from exhaustion-induced cytokine production defects. Importantly, we demonstrate that this degree of inhibition was modest enough to allow for some control of tumor cell growth during the coincubation, supporting the notion of there being an optimal balance of positive and negative signals for therapeutic NK cells that mitigates exhaustion while maintaining clinical efficacy. However, due to the modest differences in functional readouts and limited breadth of experiments in this section, these data mainly serve as proof of principle for this “signal-balancing” hypothesis. Further research, specifically with in vivo models studying this concept, must be completed to substantiate immediate clinical relevance. Nevertheless, these data highlight an understudied role of inhibitory signaling in maintaining NK cell responsiveness during an immune response to cancer and warrant further investigation.

In light of these data, it is tempting to speculate on the relevance of this work to the efficacy of NK cell–targeted checkpoint blockade, which aims to block the interaction of NK cell inhibitory receptors with their ligands on tumor cells and other subsets within the tumor microenvironment. In general, clinical trials testing the efficacy of checkpoint blockade on NK cells have generated mixed results, with several being prematurely terminated due to lack of efficacy (31, 32). In one prominent study (ClinicalTrials.gov NCT01248455), NK cells became hypofunctional following administration of IPH2101, a monoclonal antibody targeting KIR2D, an inhibitory receptor expressed on NK cells. Here, Fc γ receptor I monocytes were found to bind the therapeutic antibody and use it to remove KIR from the surface of NK cells. It was hypothesized that this deprived NK cells of a developmental process called education; although not fully understood, this is a process whereby inhibitory signaling endows NK cells with full effector potential. We do not believe we are providing education signals to NK cells in our model, but rather signals that mitigate exhaustion. Therefore, there are likely distinct mechanisms that govern the multiple routes to resistance to checkpoint blockade therapy. Given our data indicating that overstimulating NK cells without concomitant inhibitory signaling, which typically provides negative feedback to such stimuli, results in exhaustion, it is possible that NK cells subjected to checkpoint blockade in vivo encounter similar consequences. Currently, several active clinical trials are testing anti-NKG2A blockade in patients with cancer (ClinicalTrials.gov NCT02671435, NCT02643550, NCT03822351, NCT03794544), with one that has published results reporting a 27.5% overall response rate when administered in combination with cetuximab to patients with squamous cell carcinoma of the head and neck (33). As NK cell–targeting cancer therapies evolve, it will be important to keep in mind the ramifications of prolonged, unbalanced signaling that antitumor NK cells face. Indeed, many immunotherapeutic products intended to enhance NK cell tumor targeting and serial killing (e.g., mAb therapy, engager molecules, chimeric antigen receptor-NK cells) will rely on such stimulation. Therefore, further research into therapeutic modalities that prevent exhaustion are crucially needed.

## Methods

### NK cell isolation.

Peripheral blood samples were obtained from the Memorial Blood Bank (Minneapolis, Minnesota, USA). Isolation of PBMCs was carried out via density gradient centrifugation using Ficoll-Paque (GE Healthcare, now Cytiva). NK cells were enriched using NK cell negative selection enrichment kits (StemCell Technologies). Immediately following enrichment, NK cells were plated for assays in either B0 media (DMEM plus Ham’s F12 medium, 2:1, supplemented with 10% heat-inactivated human AB sera; 1% penicillin-streptomycin; 25 mM 2-ME; and 0.5 mg/mL sodium selenite) or RPMI-1640 supplemented with 10% fetal bovine serum (Gibco).

### Cell culture.

K-562 (ATCC, CCL-243), P815 (ATCC, TIB-64), and HL-60-Luc2 (ATCC, CCL-240-LUC2) cell lines were cultured in RPMI-1640 supplemented with 10% fetal bovine serum and 1% penicillin-streptomycin.

### In vitro exhaustion assays.

Receptor agonists were adsorbed onto tissue culture plates for 24 hours prior to NK cell harvesting and plating. A total of 300 μL of PBS containing 5 μg/mL anti-NKp46 (R&D Systems; catalog MAB1850), 1.25 μg/mL recombinant MICA Fc-chimera (R&D Systems; catalog 1300-MA), and 1.25 μg/mL recombinant MICB Fc-chimera (R&D Systems; catalog 1599-MB) was added to 24-well plates and incubated at 4°C overnight. For the unstimulated condition, 300 μL of isotype solution (5 μg/mL; IgG2b) in PBS was added in parallel. After 24 hours, plates were washed twice with PBS and NK cells were added; NK cells were counted and resuspended in B0 medium supplemented with 1 ng/mL IL-15 (R&D Systems) at a density of 1 × 10^6^ cells/mL. Then 1 mL (1 × 10^6^ NK cells) was added to each well of the previously prepared 24-well plates. After 3 or 7 days, NK cells were harvested, washed with PBS, counted, and used for various experiments. For exhaustion assays spanning 7 days, NK cells were harvested, washed, and replated onto identical plates on day 3.

### In vitro exhaustion mitigation assays.

Receptor agonists were adsorbed onto tissue culture plates for 24 hours prior to NK cell harvesting and plating. A total of 300 μL of the following PBS solutions were added to 24-well plates and incubated at 4°C overnight: anti-NKp46 (5 μg/mL; [R&D Systems; catalog MAB1850]), recombinant MICA Fc-chimera (1.25 μg/mL [R&D Systems; catalog 1300-MA]), recombinant MICB Fc-chimera (1.25 μg/mL [R&D Systems; catalog 1599-MB]), and isotype (20 μg/mL IgG2b; [R&D Systems catalog MAB004]); anti-NKp46 (5 μg/mL; [R&D Systems catalog MAB1850]), recombinant MICA Fc-chimera (1.25 μg/mL [R&D Systems; catalog 1300-MA]), recombinant MICB Fc-chimera (1.25 μg/mL [R&D Systems; catalog 1599-MB]), and anti-NKG2A (20 μg/mL; [Beckman Coulter catalog IM2750]); or isotype only (25 μg/mL IgG2b [R&D Systems catalog MAB004]). After 24 hours, plates were washed twice with PBS and NK cells were added; NK cells were counted and resuspended in B0 medium supplemented with 1 ng/mL IL-15 (R&D Systems) at a density of 1 × 10^6^ cells/mL. NK cells were added (1 × 10^6^ cells/well) to each well of the previously prepared 24-well plates. After 7 days (including replating at day 3 as described previously) NK cells were harvested, washed with PBS, counted, and used for various experiments.

### NK cell function assays.

Unstimulated (1 ng/mL IL-15) and stimulated (1 ng/mL IL-15 plus MICA/MICB and anti-NKp46 stimulation) NK cells were cocultured for 4 hours with K-562 tumor targets at 2:1 E/T ratio (500,000:250,000). Anti–CD107a-FITC antibody (BioLegend catalog 328606) was added at the start of incubation, followed by GolgiStop and GolgiPlug (BD Biosciences) after the first hour of incubation. Cells were washed with PBS, stained with Live/Dead fixable viability stain (Thermo Fisher Scientific), stained with surface antibodies, fixed, permeabilized, and stained intracellularly with anti–IFN-γ–BV650 (BioLegend catalog 502538) and anti–TNF-α–BV421 (BioLegend catalog 502932). An LSRII (BD) was used for flow cytometric analyses.

### Flow cytometry.

Fluorochrome-conjugated antibodies were purchased from BioLegend (CD3 [catalog 317330], CD56 [catalog 362510], NKG2D [catalog 320806], CD96 [catalog 338418], granzyme B [catalog 515408], perforin [catalog 308112], IFN-γ [catalog 502538], TNF-α [catalog 502932], CD107a [catalog 328606], TIM-3 [catalog 345018], LAG-3 [catalog 369320], Ki67 [catalog 350515], human CD45 [catalog 304041], mouse CD45 [catalog 103149]); BD Biosciences (anti-CD3 [catalog 562406], CD16 [catalog 560717]); R&D Systems (TIGIT [catalog FAB7898A], TRAIL [catalog FAB687G]); Invitrogen (Fas-L [catalog 12-9919-42]; and Miltenyi Biotec (PD-1 [catalog 130-117-384], isotype IgG2b-PE [catalog 130-092-215], NKG2A [catalog 130-113-563]).

### IncuCyte live cell imaging.

Target cells (K-562 or P815; see note below) were stained with CellTrace Far Red (Thermo Fisher Scientific) according to the manufacturer’s protocol and immobilized on poly-l-ornithine-coated (MilliporeSigma) clear 96-well flat-bottom plates (1 × 10^4^ cells/well). NK cells were added to each well (2 × 10^4^) for an E/T ratio of 2:1. All cocultures were performed in B0 media supplemented with caspase-3/7 reagent (Essen BioScience). The viable target cells per well were imaged hourly over 24 hours using the IncuCyte Live Cell Analysis System (Essen BioScience). Live cell numbers were quantified using IncuCyte Zoom or S3 software and normalized to the number of live cells in the target only control group. Note: Following staining, but before plating, P815 cells were incubated for 30 minutes at room temperature in B0 media containing either anti-NKp30 (1 μg/mL; BioLegend [catalog 325223]) and isotype (1 μg/mL; R&D Systems [catalog MAB004]), anti-NKp30, and anti-NKG2A (1 μg/mL; Beckman Coulter [catalog IM2750]), or isotype alone (1 μg/mL IgG2b plus 1 μg/mL IgG1; BioLegend [catalog 400124]). Following incubation, cells were washed with PBS and plated as described above.

### Agilent Seahorse metabolic analysis.

Control (isotype stimulated), exhaustion (isotype + MICA/MICB + anti-NKp46 stimulated), and NKG2A-tempered (anti-NKG2A + MICA/MICB + anti-NKp46 stimulated) cells were resuspended in Seahorse XF Assay Medium (Agilent Technologies). A total of 1 × 10^6^ cells/well were immobilized with poly-l-lysine (MilliporeSigma). The ECAR and the OCR were measured (pmoles/min) in real time in an Xfe24 analyzer after injection of glucose (10 mM), oligomycin (1 μM), FCCP (1 μM) plus sodium pyruvate (1 mM), and rotenone plus antimycin A (0.5 μM). SRC was calculated from the change from basal oxygen consumption, after addition of glucose, to maximal oxygen consumption, after addition of FCCP. Glycolytic capacity was calculated from the difference between the maximum ECAR following oligomycin injection and the basal ECAR. Glycolytic reserve was calculated from the difference between the maximum ECAR following oligomycin injection and ECAR following glucose injection.

### In vivo mouse study.

NSG mice (Jackson Labs) were sublethally irradiated (275 cGy), and half were xenografted (i.v.) with 750,000 firefly luciferase–expressing HL-60 human acute promyelocytic leukemia cells (day –3). At day 0, tumor-bearing and non-tumor-bearing mice were given (i.v.) 1 × 10^6^ NK cells from 7-day incubations with plate-bound agonists as described previously. On that day, 2 μg IL-15 (National Cancer Institute, NIH) was injected (i.p.) per mouse then twice per week for the remainder of the experiment to induce basal maintenance of the NK cells. Retro-orbital bleeds, 100 μL, were carried out at day 14 to assess human cell content. Mice were injected with 100 μL of 30 mg/mL luciferin substrate 10 minutes prior to imaging and then anesthetized via inhalation of isoflurane gas. The presence of tumor cells by BLI was assessed using the Xenogen IVIS imaging system and analyzed with Living Image 2.5 software (Caliper Life Science).

### P815-mediated exhaustion assays.

P815 cells were harvested, counted, and irradiated (X-RAD 320, 1000 cGy). Following irradiation, P815 cells were coated with the same antibody treatments as described above in *IncuCyte live cell imaging*. NK cells were coincubated with P815 at an E/T ratio of 2:1 (400,000 NK cells: 200,000 P815) for 5 days in a 96-well plate, at which time cells were stimulated with PMA and ionomycin (25 ng/mL, 1 μg/mL, respectively) for 4 hours. After stimulation, cells were fixed, and cytokine production was assessed via flow cytometry.

### Mass CyTOF.

After harvesting, cells were counted and viability was measured using trypan blue exclusion. Two hundred thousand cells from each donor were aliquoted into 5 mL polystyrene U-bottom tubes for barcoding and CyTOF staining. Cells were stained with cisplatin (Fluidigm product 201064), followed by barcoding using the Cell-ID 20-Plex Pd Barcoding Kit (Fluidigm product 201060). After barcoding, all cells were combined into a single 5 mL polystyrene U-bottom tube and incubated in the surface marker antibody cocktail for 30 minutes at 4°C. Following surface staining, cells were then fixed using 2% paraformaldehyde. For intracellular staining, cells were permeabilized by incubation with Triton X 0.1% for 5 minutes at room temperature, followed by incubation with intracellular antibody cocktail for 30 minutes at 4°C. Stained cells were then incubated overnight with Cell-ID Intercalator (Fluidigm product 201192A). The following morning, cells were washed and run on the CyTOF 2 instrument. Wash steps were completed using Maxpar PBS (Fluidigm product 201058), Maxpar Cell Staining Buffer (Fluidigm product 201068), or Milli-Q water (MilliporeSigma) at 1600 rpm for 4 minutes. A list of all antibodies used for CyTOF can be found in Supplemental Table 1.

### Statistics.

All statistical tests (noted in figure legends) were performed and analyzed with GraphPad Prism software. Two-tailed *t* tests were used for comparisons of 2 groups. One-way ANOVA was used for comparisons of 3 or more groups. All error bars represent the mean ± SD, save for Figure 5C, where they represent SEM. Statistical significance was determined with the following thresholds: *P ≤ 0.05; **P < 0.01; ***P < 0.001; ****P < 0.0001.

### Study approval.

Blood from healthy donors was obtained after receipt of written informed consent at Memorial Blood Bank. Use of PBMCs from donors was approved by the Committee on the Use of Human Subjects in Research at the University of Minnesota (IRB 9709M00134) in accordance with the Declaration of Helsinki. Animal studies were conducted in accordance with a protocol reviewed and approved by the University of Minnesota (IACUC 1506-A32639).

## Author contributions

Scientific concepts and research study design were conceived by JAM, MF, and JSM. Experiments and data acquisition were primarily conducted by JAM. Mouse experiments were conducted by DS, RH, LB, CS, and JAM. CyTOF experiments were performed by PH. The manuscript was written by JAM, MF, and JSM.

## Supplementary Material

Supplemental data

## Version 1 06/21/2022

In-Press Preview

## Version 2 08/08/2022

Electronic publication
