# Supplementary material for: Balanced engagement of activating and inhibitory receptors mitigates human NK cell exhaustion
Source: JCI Insight. 2022 Aug 8;7(15):e150079. doi: 10.1172/jci.insight.150079 (PMC9462468; doi:10.1172/jci.insight.150079)
Supplement: Supplemental data [file jciinsight-7-150079-s007.pdf]

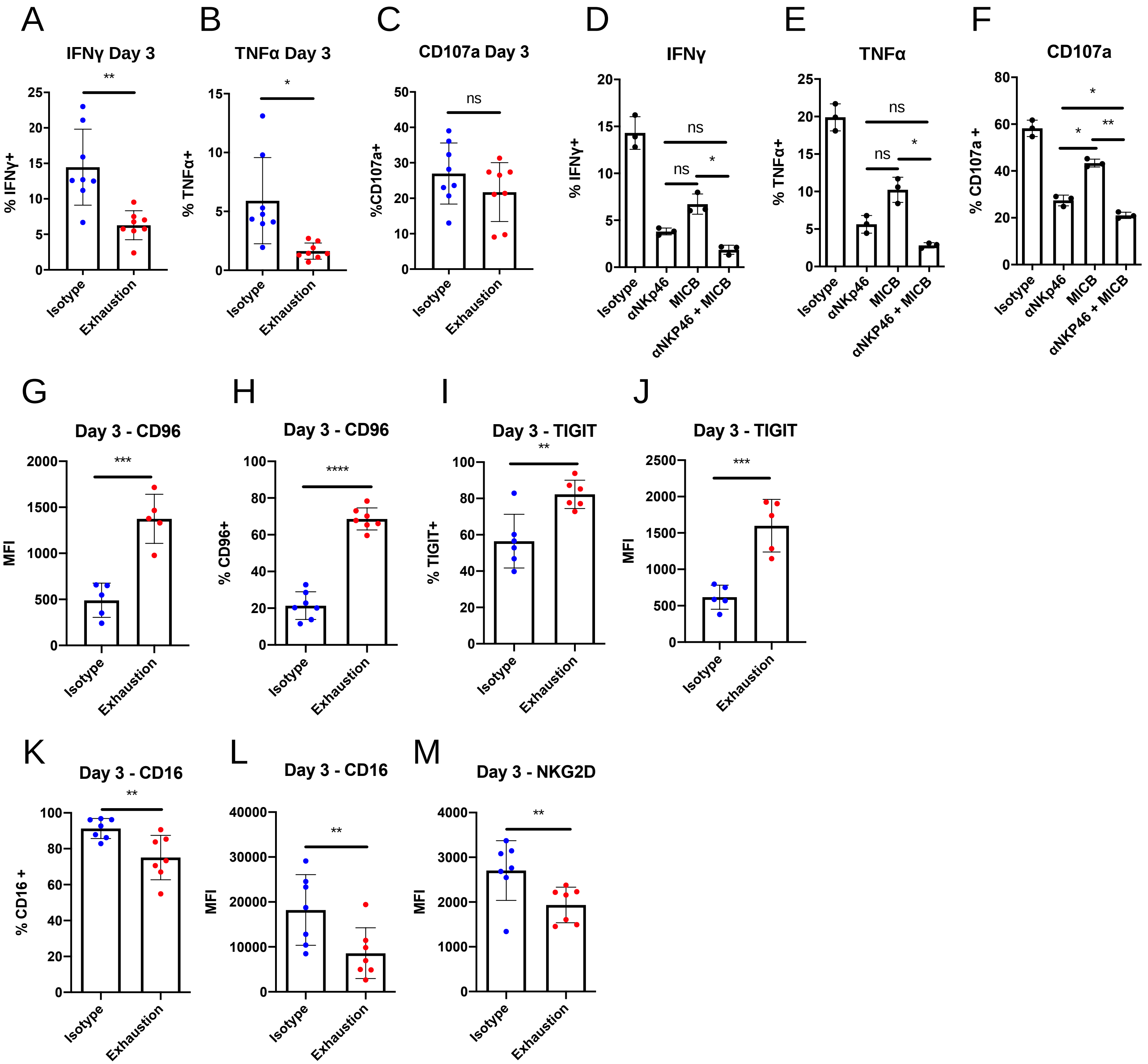

SUPPLEMENTARY FIGURE 1. (**A**-**C**) NK cells harvested from isotype-coated and exhaustion plates (day 3) were incubated with K562 targets for four hours (E/T: 2:1). Production of IFNγ (A) (*n*=8) and TNFα (B) (*n*=8) and degranulation (CD107a [C]) (*n*=8) were measured via flow cytometry. (**D**-**F**) NK cells were stimulated through NKp46 and NKG2D independently and simultaneously for 7 days. Production of IFNγ (D) (*n*=3) and TNFα (E) (*n*=3) and degranulation (CD107a [F]) (*n*=3) were measured via flow cytometry. (**G-M**) On day 3 of stimulation, flow cytometry was used to stain for several receptors. Percent of parent and MFI are included where indicated. CD96 (G-H), TIGIT (I-J), CD16 (K-L), NKG2D (M); (*n*=7); $*P \leq 0.05$; $**P < 0.01$; $***P < 0.001$; $****P < 0.0001$ ns, not significant.

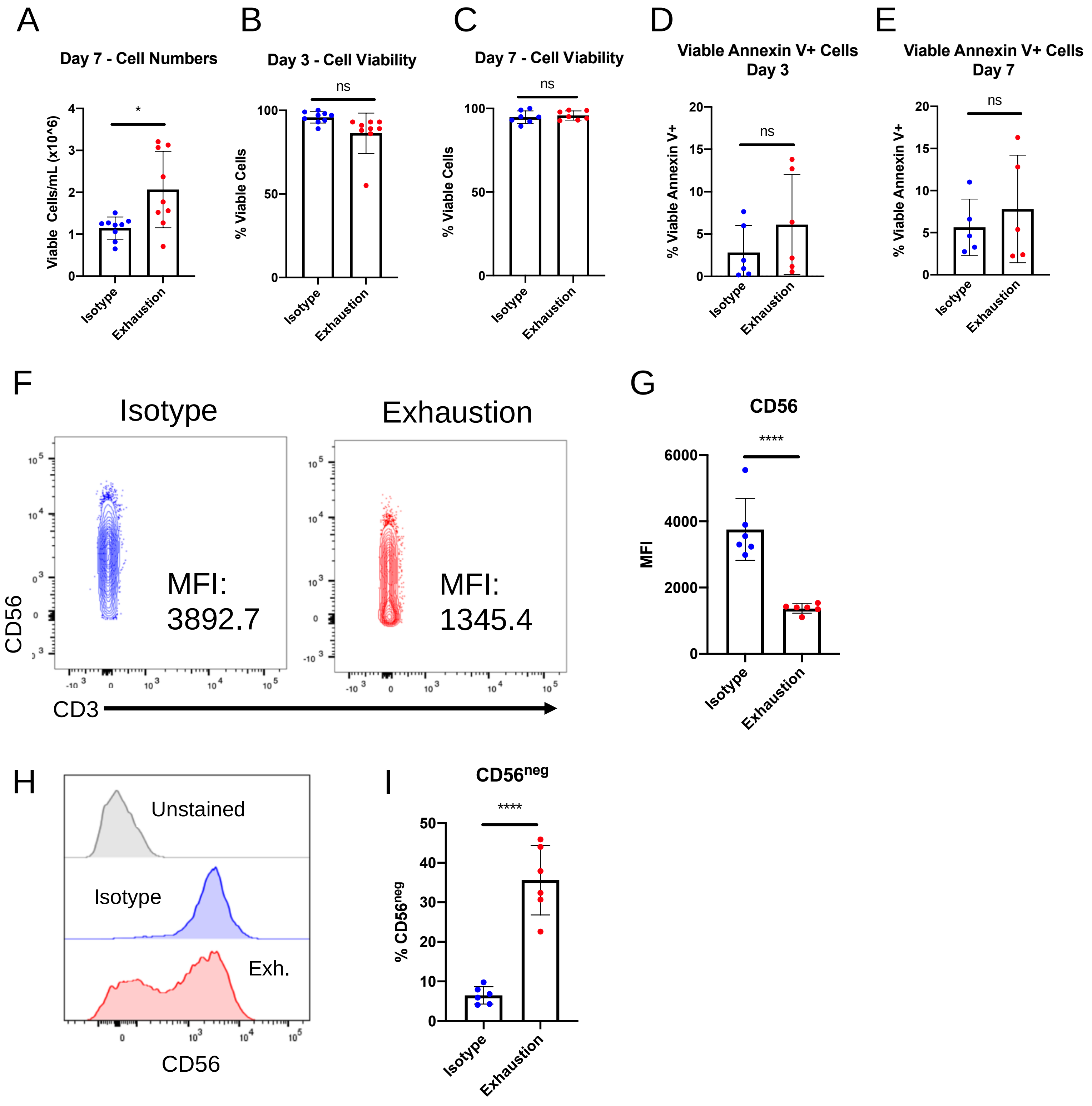

SUPPLEMENTARY FIGURE 2. (**A**) Viable cells were quantified at day 7 using the Beckman Coulter ViCell counter. (Starting cell density = 1x10^6 cells/mL, *n*=9.) Paired *t* tests were used for comparisons. *$P \leq 0.05$. (**B**-**C**) Viability of NK cells harvested from isotype-coated and exhaustion plates (harvested at days 3 [B] [*n*=9] and 7 [C] [*n*=7]) was assessed via Trypan blue uptake. (**D-E**) NK cells harvested from isotype-coated

and exhaustion plates (days 3 [D] [*n*=6] and 7 [E] [*n*=5]) were stained with Annexin V to assess apoptosis. (Analysis was performed via flow cytometry.) (**F**) Representative contour plots of CD56 expression for isotype (blue) and exhausted (red) cells. Analysis was performed via flow cytometry: NK cells were live, CD3- CD56+. (**G**) Quantification of pooled results (*n*=7). (F) Histogram of CD56 expression. (I) Quantification of pooled results (*n=6*) ***P < 0.001; ****P < 0.0001 ns, not significant.

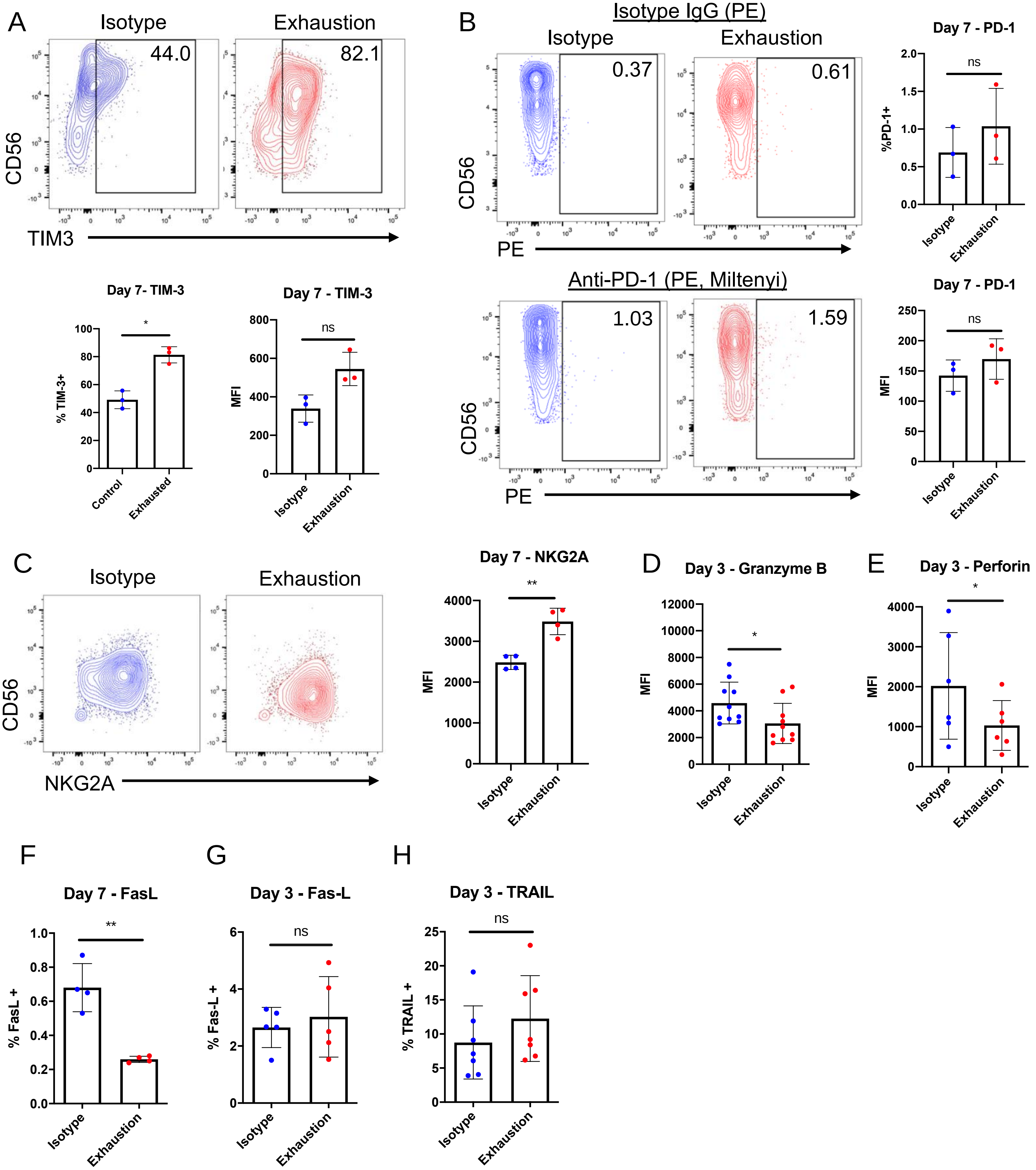
A
Isotype
Exhaustion
44.0
82.1
CD56
TIM3
Day 7- TIM-3
% TIM-3+
Control
Exhausted
Day 7 - TIM-3
MFI
Isotype
Exhaustion
ns
B
Isotype IgG (PE)
Isotype
Exhaustion
0.37
0.61
CD56
PE
Anti-PD-1 (PE, Miltenyi)
1.03
1.59
Day 7 - PD-1
%PD-1+
ns
Day 7 - PD-1
MFI
ns
C
Isotype
Exhaustion
CD56
NKG2A
Day 7 - NKG2A
MFI
D
Day 3 - Granzyme B
MFI
E
Day 3 - Perforin
MFI
F
Day 7 - FasL
% FasL +
G
Day 3 - Fas-L
% Fas-L +
ns
H
Day 3 - TRAIL
% TRAIL +
ns

SUPPLEMENTARY FIGURE 3. (**A-H**) NK cells were harvested from isotype-coated and exhaustion plates and the expression of various receptors was analyzed via flow cytometry: TIM3, Day 7 (A) (*n*=3) representative dot plots, MFI data, and % of parent included; PD-1, Day 7 (B) (*n*=3) PE-conjugated isotype control, representative dot plots, MFI data, and % of parent included; NKG2A (C) (*n*=4) representative dot plots and MFI data included; Granzyme B, Day 3 (D) (*n*=10); Perforin, Day 3 (E) (*n*=6); Fas-L, Day 7 and 3 (F-G) (*n*=4, *n*=5); TRAIL, Day 3 (H) (*n=8*). *$P \leq 0.05$; **$P < 0.01$; ns, not significant.

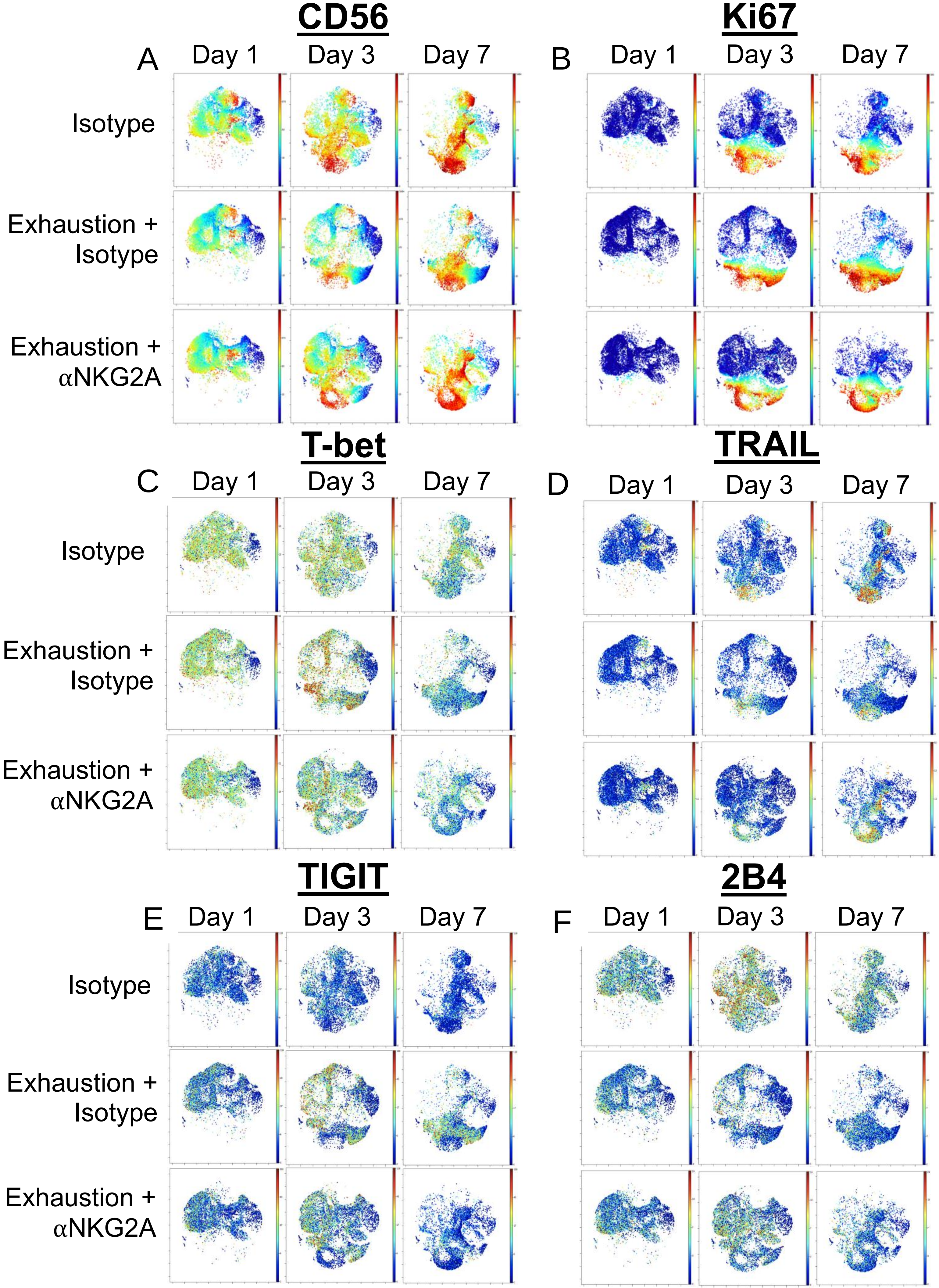

CD56
A
Day 1
Day 3
Day 7
Isotype
Exhaustion + Isotype
Exhaustion + αNKG2A
Ki67
B
Day 1
Day 3
Day 7
T-bet
C
Day 1
Day 3
Day 7
Isotype
Exhaustion + Isotype
Exhaustion + αNKG2A
TRAIL
D
Day 1
Day 3
Day 7
TIGIT
E
Day 1
Day 3
Day 7
Isotype
Exhaustion + Isotype
Exhaustion + αNKG2A
2B4
F
Day 1
Day 3
Day 7

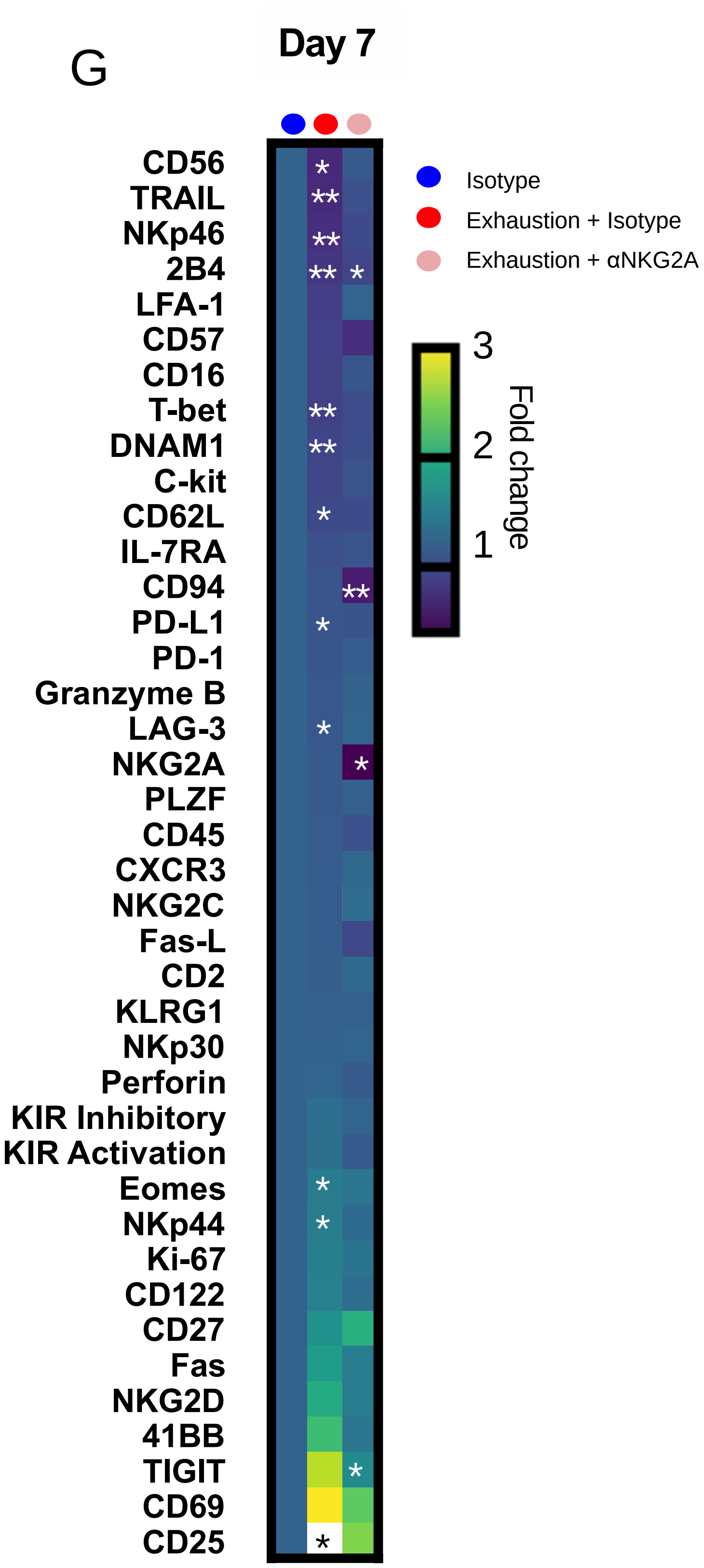

SUPPLEMENTAL FIGURE 4. (**A-G**) At day 7 of stimulation, cells from three donors were analyzed via mass cytometry (CyTOF). viSNE analysis was performed for several markers: CD56 (A), Ki67 (B), T-bet (C), TRAIL (D), TIGIT (E), and 2B4 (F). Heatmap (G) indicates fold change relative to isotype mean metal intensity (MMI). * $P \leq 0.05$; ** $P < 0.01$; data that is not significant was left unlabeled (n=3).

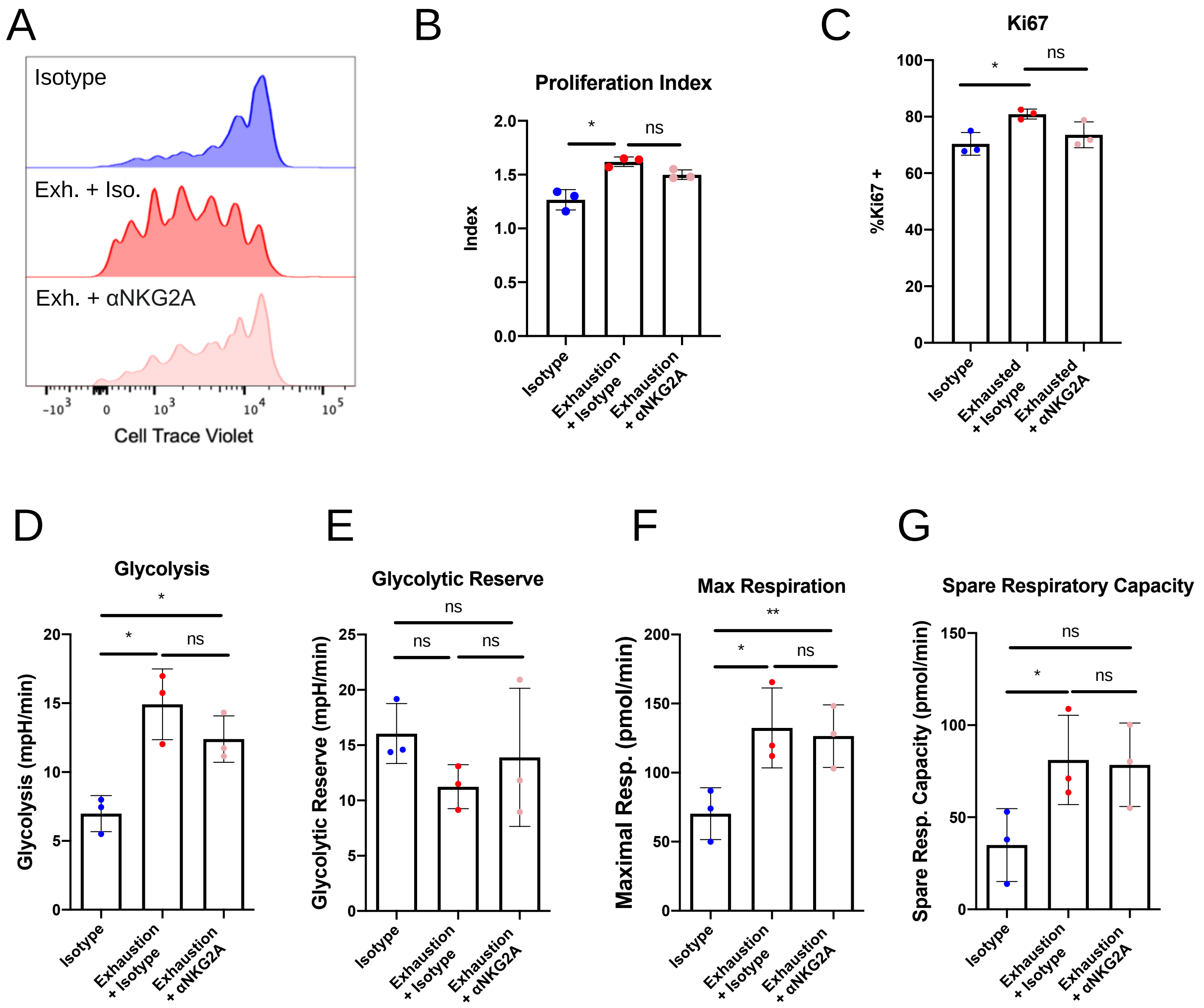

SUPPLEMENTAL FIGURE 5. (**A**) NK cells were stained with Cell Trace Violet and cultured on stimulatory plates for 7 days, as previously described. Cells were analyzed via flow cytometry 7 days later. (**B**) Proliferative indices were calculated using FlowJo V10 (**C**) NK cells were stained with Ki67 and analyzed via flow cytometry (*n*=3). (**C**) NK cells were harvested at day 7 and stained for Ki67 (*n*=3). (**D-G**) NK cells were immobilized on tissue culture plates and subjected to live-cell metabolic assays using Agilent's Seahorse XFe24 Analyzer. Extracellular acidification rates (ECAR) were measured following injection of glucose (G), oligomycin (O), FCCP (F), and Antimycin A + Rotenone (A+R). Oxygen consumption rates were measured following injection of glucose (G), oligomycin (O), FCCP (F), and Antimycin A + Rotenone (A+R). Graphical representations of glycolysis (D), glycolytic reserve (E), maximal respiration (F), and spare respiratory capacity (G) are pictured (*n*=3) ONE way ANOVA was used for comparisons. *$P \leq 0.05$; **$P < 0.01$; ns, not significant.

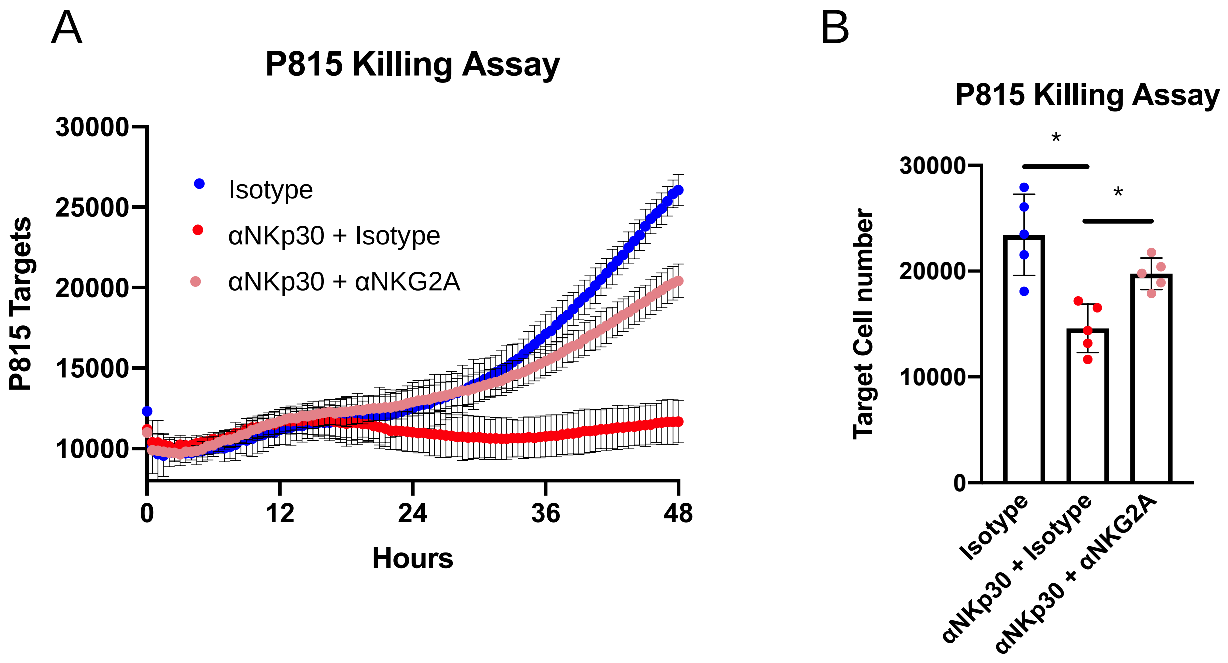

SUPPLEMENTAL FIGURE 6. (**A**) P815 targets were labelled with Cell trace Far Red and coated with either anti-NKp30 and anti-NKG2A or anti-NKp30 and isotype IgG. P815 cells coated in isotype alone served as a negative control. NK cells were incubated with P815 target cells (E/T 2:1) and target cell growth was tracked using the Incucyte live cell imaging platform. (**B**) The number of target cells remaining after 48 hours was quantified for every condition and E/T ratio (*n*=5). $^*P \leq 0.05$.

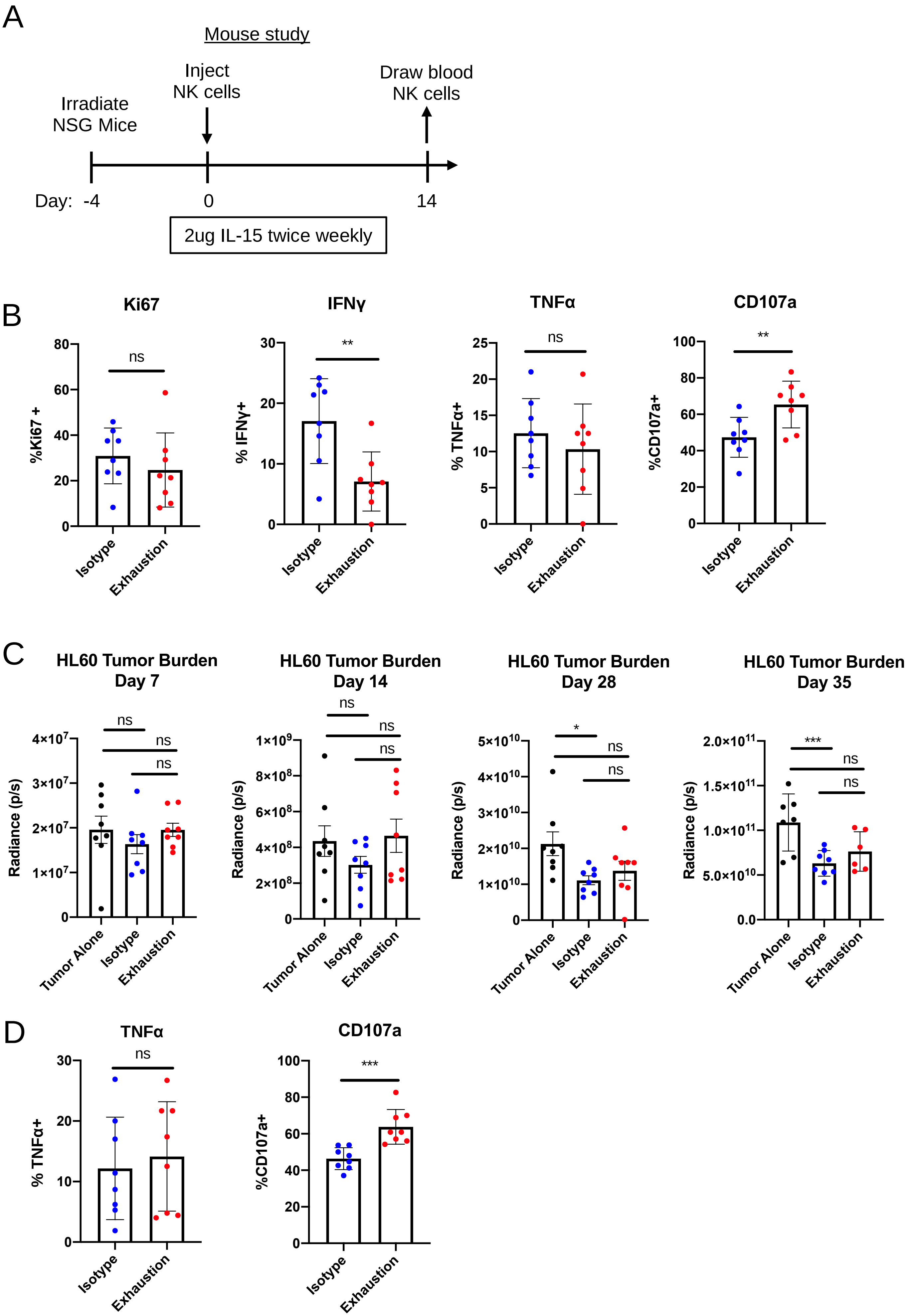

A
Mouse study
Irradiate NSG Mice
Inject NK cells
Draw blood NK cells
Day: -4
0
14
2ug IL-15 twice weekly
B
Ki67
IFNγ
TNFα
CD107a
%Ki67 +
% IFNγ+
% TNFα+
%CD107a+
ns
**
ns
**
Isotype
Exhaustion
C
HL60 Tumor Burden Day 7
HL60 Tumor Burden Day 14
HL60 Tumor Burden Day 28
HL60 Tumor Burden Day 35
Radiance (p/s)
Tumor Alone
Isotype
Exhaustion
ns
*
***
D
TNFα
CD107a
% TNFα+
%CD107a+
ns
***
Isotype
Exhaustion

SUPPLEMENTAL FIGURE 7. (**A**) Schematic representing experimental design: sublethally irradiated NSG mice were injected (i.v.) with $1x10^6$ NK cells. NK cells had been incubated for 7 days on plates with either isotype IgG (control) or anti-NKp46 and MICA/B as previously described. (**B**) 14 days post NK cell injection in the non-tumor model, blood was drawn, and NK cells were restimulated with K562 leukemia cells for 4 hours as previously described. Ki67, IFNγ, TNFα, and CD107a expression was assessed via flow cytometry. NK cells were CD45h+ CD56+ CD3-. Paired *t* tests were used for comparisons (*n*=8). $^{**}P < 0.01$; ns, not significant. (**C**) Tumor burden at several time points was measured via BLI. One way ANOVA was used for comparisons. $^{*}P < 0.05$; $^{***}P < 0.001$; ns, not significant (*n*=8). (**D**) 14 days post NK cell injection in the HL60 tumor model, blood was drawn, and NK cells were restimulated with K562 leukemia cells for 4 hours as previously described. TNFα and CD107a expression was assessed via flow cytometry. NK cells were CD45h+ CD56+ CD3-. Paired *t* tests were used for comparisons (*n*=8). $^{***}P < 0.001$; ns, not significant.

A

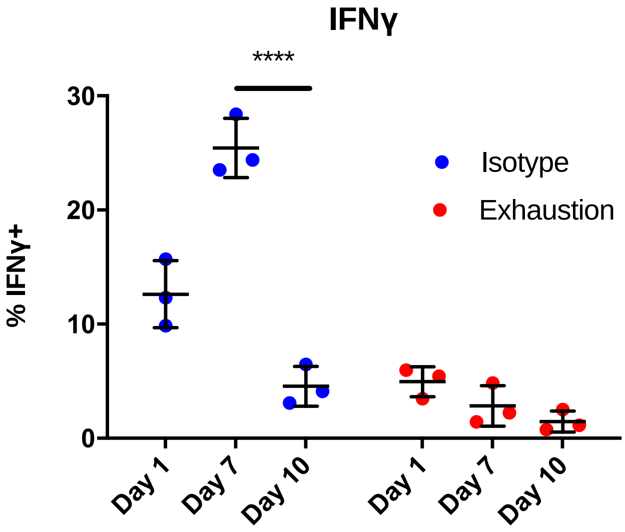

SUPPLEMENTARY FIGURE 8. (**A**) NK cells harvested from isotype-coated and exhaustion plates (days 1, 7, and 10) were incubated with K562 targets for four hours (E/T: 2:1). IFNγ production was measured via flow cytometry. Paired *t* tests were used for comparisons (*n*=3). ****$P < 0.0001$.

Marker	Metal	Catalog no.	Clone
CD3	141Pr	3141019B	UCHT1
KIR Activating Pool	142Nd	201142A	KIR2DL1(143211)
C-Kit	143Nd	3143001B	104D2
CD69	144Nd	3144018B	FN50
KIR Inhibitory Pool	145Nd	201145A	KIR2DS1 (1127B)
CD8a	146Nd	3146001B	RPA-T8
Fas Ligand	147Sm	201147A	NOK-1
CD274 (PD-L1)	148Nd	3148017B	29E.2A3
CD25 (IL-2R)	149Sm	3149010B	2A3
PLZF	150Nd	201150A	6318100
CD2	151Eu	3151003B	TS1/8
CD95/Fas	152Sm	3152017B	DX2
CD62L (L-selectin)	153Eu	3153004B	DREG-56
CD16	154Sm	3154016B	MBSA43
CD27	155Gd	3155001B	L128
CXCr3	156Gd	3156004B	G025H7
CD137/4-1BB	158Gd	3158013B	4B4-1
CD337 (NKp30)	159Tb	3159017B	Z25
NKG2C	160Gd	201160A	2098A
Eomes	161Dy	201161A	644730
CD335 (NKp46)	162Dy	3162021B	BAB281
CD56 (NCAM)	163Dy	3163007B	NCAM 16.2
TRAIL	164Dy	201164A	RIK2
CD223/LAG-3	165Ho	3165037B	11C3C65
CD314 (NKG2D)	166Er	3166016B	ON72
NKp44	167Er	201167A	P-448
Ki-67	168Er	3168007B	B56
CD159a (NKG2A)	169Tm	3169013B	Z199
CD122	170Er	3170004B	Tu27
CD226 DNAM-1	171Yb	3171013B	DX11
CD57	172Yb	3172009B	HCD57
Granzyme B	173Yb	3173006B	GB11
CD279 (PD-1)	174Yb	3174020B	EH12.2H7

Perforin	175Lu	3175004B	B-D48
CD127 (IL-7Ra)	176Yb	3176004B	A019D5
TIGIT	209Bi	3209002B	MBSA43
CD45	89Y	3089003B	HI30
CD94	111CD	201111A	DX22
LFA-1	112CD	201112A	M24
KLRG-1	113CD	201113A	14C2A07
2B4	114CD	201114A	C1.7
T-bet	116CD	201116A	4B10

SUPPLEMENTARY TABLE 1. Antibodies used for Mass Cytometry by time-of-flight (CyTOF). Marker, metal tag, catalog number, and clone ID for each antibody are listed.
